# Genetic Polymorphisms of the Human PNPLA3 Gene Are Strongly Associated with Severity of Non-Alcoholic Fatty Liver Disease in Japanese

**DOI:** 10.1371/journal.pone.0038322

**Published:** 2012-06-14

**Authors:** Takahisa Kawaguchi, Yoshio Sumida, Atsushi Umemura, Keitaro Matsuo, Meiko Takahashi, Toshinari Takamura, Kohichiroh Yasui, Toshiji Saibara, Etsuko Hashimoto, Miwa Kawanaka, Sumio Watanabe, Sumio Kawata, Yasuharu Imai, Miki Kokubo, Toshihide Shima, Hyohun Park, Hideo Tanaka, Kazuo Tajima, Ryo Yamada, Fumihiko Matsuda, Takeshi Okanoue for the Japan Study Group of Nonalcoholic Fatty Liver Disease (JSG-NAFLD)

**Affiliations:** 1 Center for Genomic Medicine, Kyoto University Graduate School of Medicine, Kyoto, Japan; 2 Institut National de la Sante et de la Recherche Medicale (INSERM) Unite U852, Kyoto University Graduate School of Medicine, Kyoto, Japan; 3 Center for Digestive and Liver Diseases, Nara City Hospital, Nara, Japan; 4 Center of Gastroenterology and Hepatology, Saiseikai Suita Hospital, Suita, Japan; 5 Division of Epidemiology and Prevention, Aichi Cancer Center, Nagoya, Japan; 6 Department of Disease Control and Homeostasis, Kanazawa University, Graduate School of Medical Science, Kanazawa, Japan; 7 Department of Molecular Gastroenterology and Hepatology, Graduate School of Medical Science, Kyoto Prefectural University of Medicine, Kyoto, Japan; 8 Department of Gastroenterology and Hepatology, Kochi Medical School, Kochi, Japan; 9 Department of Internal Medicine and Gastroenterology, Tokyo Women’s Medical University, Tokyo, Japan; 10 Center of Liver Diseases, Kawasaki Hospital, Kawasaki Medical School, Okayama, Japan; 11 Department of Gastroenterology, Juntendo University School of Medicine, Tokyo, Japan; 12 Department of Gastroenterology, Yamagata University School of Medicine, Yamagata, Japan; 13 Department of Internal Medicine, Ikeda Municipal Hospital, Ikeda, Japan; Wageningen University, The Netherlands

## Abstract

**Background:**

Nonalcoholic fatty liver disease (NAFLD) includes a broad range of liver pathologies from simple steatosis to cirrhosis and fibrosis, in which a subtype accompanying hepatocyte degeneration and fibrosis is classified as nonalcoholic steatohepatitis (NASH). NASH accounts for approximately 10–30% of NAFLD and causes a higher frequency of liver-related death, and its progression of NASH has been considered to be complex involving multiple genetic factors interacting with the environment and lifestyle.

**Principal Findings:**

To identify genetic factors related to NAFLD in the Japanese, we performed a genome-wide association study recruiting 529 histologically diagnosed NAFLD patients and 932 population controls. A significant association was observed for a cluster of SNPs in *PNPLA3* on chromosome 22q13 with the strongest *p*-value of 1.4×10^−10^ (OR = 1.66, 95%CI: 1.43–1.94) for rs738409. Rs738409 also showed the strongest association (*p* = 3.6×10^−6^) with the histological classifications proposed by Matteoni and colleagues based on the degree of inflammation, ballooning degeneration, fibrosis and Mallory-Denk body. In addition, there were marked differences in rs738409 genotype distributions between type4 subgroup corresponding to NASH and the other three subgroups (*p* = 4.8×10^−6^, OR = 1.96, 95%CI: 1.47–2.62). Moreover, a subgroup analysis of NAFLD patients against controls showed a significant association of rs738409 with type4 (*p* = 1.7×10^−16^, OR = 2.18, 95%CI: 1.81–2.63) whereas no association was obtained for type1 to type3 (*p* = 0.41). Rs738409 also showed strong associations with three clinical traits related to the prognosis of NAFLD, namely, levels of hyaluronic acid (*p* = 4.6×10^−4^), HbA1c (*p* = 0.0011) and iron deposition in the liver (*p* = 5.6×10^−4^).

**Conclusions:**

With these results we clearly demonstrated that Matteoni type4 NAFLD is both a genetically and clinically different subset from the other spectrums of the disease and that the *PNPLA3* gene is strongly associated with the progression of NASH in Japanese population.

## Introduction

Nonalcoholic fatty liver disease (NAFLD) includes a broad range of pathologies from fatty liver (simple steatosis), steatonecrosis, and steatohepatitis to cirrhosis [1]–[3]. NAFLD often accompanies other lifestyle-related pathologies of metabolic syndrome such as diabetes mellitus, hypertension and dyslipidemia, and the number of NAFLD patients is increasing worldwide along with the escalation in the incidence of metabolic syndrome [4]. Prevalence of NAFLD is considered as approximately 8% in Japanese and 6–35% in Europeans [4], [5]. The majority of NAFLD shows simple steatosis with a good prognosis, but approximately 10–30% of NAFLD histologically diagnosed as nonalcoholic steatohepatitis (NASH) shows hepatocyte degeneration (ballooning hepatocyte), necrosis, inflammation and fibrosis, with a higher frequency of liver-related death both in Japanese and European populations [6], [7]. Insulin resistance and oxidative stress are considered to be key players in the progression of NASH [8], [9]. However, the progression of NASH has been considered to be complex involving multiple genetic factors interacting with the environment and lifestyle, because only a portion of NAFLD patients develops NASH.

The first Genome-wide association (GWA) study searching for such genetic factors identified the *PNPLA3* gene as a major genetic determinant for the predisposition to NAFLD in Hispanic, African American and European American populations according to liver fat contents [10], which was subsequently confirmed in Europeans and Asians according to liver biopsy. Association of *PNPLA3* with not only fatty liver and TG content, but also inflammation and fibrosis were shown in the subsequent studies, so *PNPLA3* may be widely associated with the development of NAFLD [11]–[13]. More recently, another GWA study reported the association of four additional genes with NAFLD in Europeans [14]. Also, a candidate gene-based approach revealed the association between NAFLD and the apolipoprotein C3 gene in Indians [15]. However, the precise role of such genes in the development of NASH still remains to be elucidated. In addition, no GWA study has been reported for Asian populations to date although the genetic components and their relative contribution may be different between ethnicities.

The Japan NASH Study Group was founded in 2008 aiming at the identification of genetic determinants predisposing to NASH in the Japanese population. Here we report the first GWA study of NAFLD in the Japanese using DNA samples of patients with liver histology-based diagnoses recruited through this multi-institutional research network.

## Results

### Genome-wide Association Analysis of NAFLD in Japanese

We conducted a GWA study using DNA samples of 543 patients with NAFLD and 942 controls. After quality controls of genotyping results (see materials and methods for details), a total of 529 patients consisting of four NAFLD subgroups according to Matteoni’s classification [2] (type1; 100, type2; 73, type3; 29, type4; 327) and 932 controls were subjected to statistical analyses (Table 1). This index pathologically classifies NAFLD according to the degree of inflammation, hepatocyte degeneration, and the existence of fibrosis and Mallory-Denk body in the liver. Genome scan results of 932 DNA samples collected for other genetic studies were used as general Japanese population controls [16]. After standard quality control procedure as described in materials and methods, genotype distributions of 484,751 autosomal SNP markers were compared between the NAFLD cases and control subjects by exact trend test. A slight inflation of *p*-values was observed by genomic control method (λ = 1.04) (Figure S1).

**Table 1:** Clinical characteristics according to the histological classification.

Phenotype	Matteoni classification of NAFLD				Control	*p-*value
	Type 1	Type 2	Type 3	Type 4		
Number of samples	100	73	29	327	932	
Sex (Male/Female)	59/41	47/26	13/16	130/197	471/461	0.0023‡
Age (year)	49.7±15.3	51.5±15.3	49.4±14.0	57.6±14.8	48.8±16.3	<0.001
Physical measurement						
BMI	26.2±4.3	27.7±4.8	27.6±3.5	27.7±5.2	–	0.054
Amount of visceral fat (cm^2^)	146.8±65.3	154.3±47.7	136.8±53.8	151.7±57.4	–	0.46
Abdominal circumscript (cm)	90.9±9.9	94.1±10.0	88.5±10.2	94.1±11.8	–	0.10
Biochemical trait						
AST (IU/L)	31.1±14.6	36.4±18.5	52.4±35.1	57.7±48.4	–	<0.001
ALT (IU/L)	48.6±30.8	62.8±47.6	81.5±46.9	74.9±48.4	–	<0.001
GGT (IU/L)	71.0±62.5	67.1±66.9	96.1±91.3	76.6±73.9	–	0.25
Albumin (g/dL)	4.5±0.4	4.4±0.3	4.5±0.3	4.3±0.4	–	<0.001
Total bilirubin (mg/dL)	0.9±0.5	0.9±0.5	0.9±0.6	0.8±0.4	–	0.063
Cholinesterase (unit)	389.1±97.0	354.3±97.2	371.1±109.9	348.9±93.2	–	<0.001
Type IV collagen 7S (ng/dL)	3.8±0.7	3.9±0.9	3.9±0.8	5.1±1.7	–	<0.001
Hyaluronic acid (ng/dL)	25.6±22.5	33.6±29.5	31.5±24.0	80.9±84.3	–	<0.001
Triglycerides (mg/dL)	151.9±73.8	154.0±92.1	166.1±86.5	161.2±85.7	–	0.23
Total cholesterol (mg/dL)	209.1±32.8	194.0±38.0	203.0±39.9	200.3±39.0	–	0.093
HbA1c (%)	6.1±1.1	5.9±1.2	6.5±1.8	6.2±1.3	–	0.13
IRI (µg/dL)	9.1±5.4	11.4±9.0	10.4±6.3	14.9±9.9	–	<0.001
FPG (mg/dL)	112.9±33.7	107.3±27.4	109.9±27.7	114.8±33.8	–	0.14
HOMA-IR	2.4±1.5	2.9±2.4	3.0±2.1	4.2±3.0	–	<0.001
hs-CRP (mg/dL)	1078.9±1407	1048.3±1185.0	865.8±658.4	1579.2±2377.9	–	0.027
Adiponectin (µg/mL)	7.4±4.4	8.5±6.6	6.6±2.6	6.9±4.3	–	0.24
Leptin (ng/mL)	9.9±7.4	9.1±6.2	11.3±9.4	12.4±7.9	–	<0.001
Ferritin (ng/mL)	145.8±101.1	176.5±134.0	271.2±307.0	208.3±180.3	–	0.027
Uric acid (mg/dL)	5.9±1.5	5.7±1.2	5.4±1.9	5.7±1.6	–	0.77
PLT (×10^4^/µL)	23.0±5.9	22.9±4.9	21.9±6.7	20.2±6.4	–	<0.001
ANA (0/1/2/3/4)	42/17/4/0/0	31/8/4/1/2	15/6/2/0/0	147/76/31/8/12	–	0.015
Clinical history						
Diabetes (NGT/IGT/DM)	36/11/34	24/7/27	12/8/7	103/35/119	–	0.45*
Hyperlipidemia (+/−)	31/68	31/42	9/20	120/206	–	0.60‡
Hypertension (+/−)	64/35	33/40	19/10	155/172	–	0.013‡
Liver biopsy feature						
Brunt grade (1/2/3)	–	–	19/3/2	149/133/44	–	<0.001‡
Brunt stage (1/2/3/4)	–	–	–	123/74/105/24	–	–
Fat droplet (1/2/3/4)	38/32/19/11	14/29/18/7	7/3/10/4	51/99/104/52	–	<0.001
Iron deposition (0/1/2/3/4)	30/14/21/10/1	24/9/12/2/1	10/5/2/2/0	132/56/29/29/11	–	0.16

Measurements are shown as mean ± standard deviation. Categorical values are shown by the count number. *P*-values are calculated by Jonckheere-Terpstra test unless otherwise stated;

‡ Chochran-Armitage trend test,

* Kruskal-Wallis test. Abbreviations used for each trait are summarized in materials and methods.

**Figure 1:**
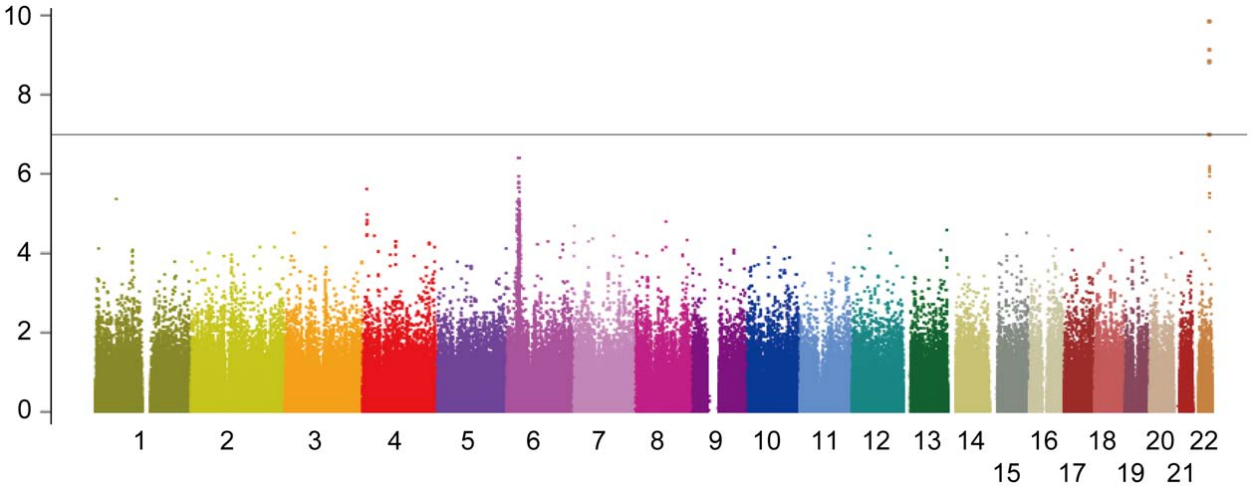
Manhattan plot of the GWA study. Association *p*-values are calculated by exact trend test and plotted along the chromosome in −log_10_ scale. The horizontal line indicates Bonferroni-adjusted significance threshold (*p* = 1.03×10^−7^).

**Figure 2:**
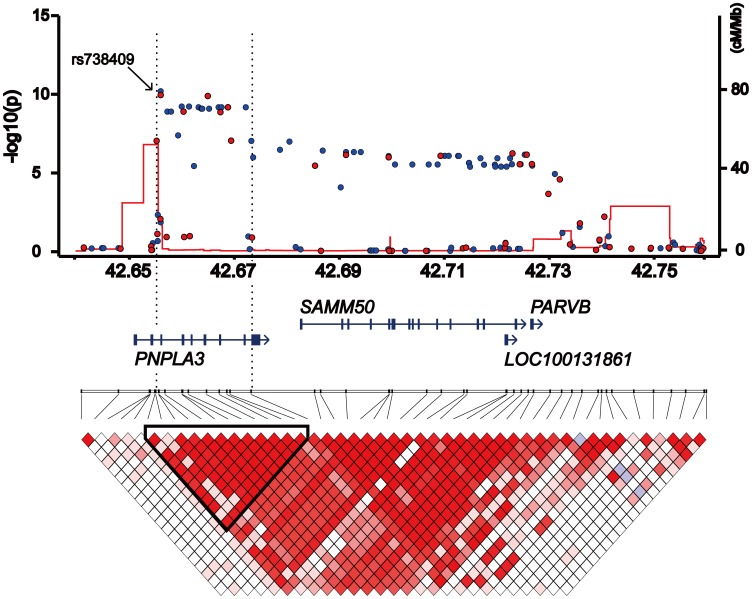
A schematic organization of the human *PNPLA3* locus at 22q13.31 with the genome scan results. *P*-values calculated by the exact trend test were plotted in –log_10_ scale. Red and blue dots indicate the *p*-values of genotyped and imputed SNPs, respectively. Local recombination rate obtained from HAPMAP release 22 is indicated by a red line plotted in cm/Mb scale. The structure and orientation of four genes in the region are shown below the plots with their transcriptional orientations according to NCBI Reference Sequence Build 36.3. LD blocks were generated according to pairwise LD estimates of the SNPs located within the region using the genome scan results. The LD block showing the strongest association is highlighted with the triangle, and the corresponding chromosomal region is represented by the dotted lines.

We identified six SNP markers located at chromosome 22q13 showing genome-wide significance (*p*<1.04×10^−7^) (Figure 1). Among them, four SNPs, namely, rs2896019, rs926633, rs2076211 and rs1010023, located in the *PNPLA3* gene and in strong linkage disequilibrium (LD) (r^2^>0.93), returned *p*-values smaller than 1×10^−9^ (*p* = 1.5×10^−10^, 7.5×10^−10^, 1.4×10^−9^ and 1.5×10^−9^, respectively) (Table 2). Rs738407 and rs3810662 also located in *PNPLA3* showed significant but weaker associations (*p* = 1.0×10^−7^ and 1.0×10^−7^, respectively) than the above four SNP markers. Rs738491, rs2073082, rs3761472, rs2235776, rs2143571 and rs6006473 were in the neighboring *SAMM50* gene which is outside of the linkage disequilibrium (LD) block where the top SNP markers were distributed (Figure 2). These markers were in moderate LD with each other (r^2^>0.42) and showed *p*-values between 3.9×10^−6^ and 6.4×10^−7^ but did not reach genome-wide significance (Table S1). Rs738409, the SNP which showed the strongest association with NAFLD in the first GWA study [10], was not included in the SNP array used in our study. This SNP was therefore genotyped using Taqman technology in the same case and control samples that were used for genome scan. Rs738409 showed the strongest association with the disease (*p* = 1.4×10^−10^, OR = 1.66, 95%CI: 1.43–1.94) among all the SNP markers examined in this study. The association remained after the correction for population stratification with EIGENSTRAT [17] (*p* = 2.3×10^−11^). Although a peak consisting of a cluster of SNPs was observed at the *HLA* locus on chromosome 6 (minimal *p*-value of 4.10×10^−7^ for rs9262639 located at the 3′ of *C6orf15* gene), the association disappeared when EIGENSTRAT was applied (*p*>1.6×10^−3^). We consider this as a result of population stratification between the cases and controls.

**Table 2:** List of the SNP markers in the *PNPLA3* locus at chromosome 22q showing genome wide significance.

		Genotyping Result and Allele Frequency of A2						Statistics		
			NAFLD					NAFLD vs. Control		Matteoni
dbSNPID	A1/A2	Control	Total	Type 1	Type 2	Type 3	Type 4	*p-*value†	OR (95%CI)	*p-*value‡
rs738407	T/C	124/447/361	46/200/283	12/51/37	10/28/35	4/14/11	20/107/200	1.0×10^−7^	1.56(1.32–1.83)	3.4×10^−5^
		(0.627)	(0.724)	(0.625)	(0.671)	(0.621)	(0.775)			
rs738409	C/G*	247/468/217	88/236/203	20/59/21	21/30/22	8/11/9	39/136/151	1.4×10^−10^	1.66(1.43–1.94)	3.6×10^−6^
		(0.484)	(0.609)	(0.505)	(0.507)	(0.518)	(0.672)			
rs2076211	C/T*	248/473/211	92/242/195	21/58/21	21/30/22	8/11/10	42/143/142	1.4×10^−9^	1.61(1.38–1.87)	3.2×10^−5^
		(0.480)	(0.597)	(0.500)	(0.507)	(0.534)	(0.653)			
rs2896019	T/G*	246/473/213	91/234/204	20/57/23	22/29/22	7/12/10	42/136/149	1.5×10^−10^	1.66(1.42–1.93)	2.6×10^−5^
		(0.482)	(0.607)	(0.515)	(0.500)	(0.552)	(0.664)			
rs1010023	T/C*	249/473/210	94/239/196	21/57/22	22/29/22	7/12/10	44/141/142	1.5×10^−9^	1.61(1.38–1.87)	6.5×10^−5^
		(0.479)	(0.596)	(0.505)	(0.500)	(0.552)	(0.650)			
rs926633	G/A*	247/474/211	93/237/199	21/56/23	22/29/22	7/12/10	43/140/144	7.5×10^−10^	1.62(1.39–1.89)	5.8×10^−5^
		(0.481)	(0.600)	(0.510)	(0.500)	(0.552)	(0.654)			
rs3810622	T*/C	330/445/157	263/208/58	40/48/12	28/29/16	14/12/3	181/119/27	1.0×10^−7^	0.64(0.55–0.75)	0.0017
		(0.407)	(0.306)	(0.360)	(0.418)	(0.310)	(0.265)			

Reference (A1) and non-reference (A2) alleles refer to NCBI Reference Sequence Build 36.3 with the effective allele marked by an asterisk. Genotyping results are shown by genotype count of A1A1/A1A2/A2A2 with allele frequency of A2 in parenthesis.

† *P*-values are calculated by exact trend test with odds ratios (OR) calculated for A2 with 95% confidence interval (CI).

‡ *P*-values are calculated by Jonckheere-Terpstra test in NAFLD patients for Matteoni type and additive model of genotype. SNPs are ordered by chromosomal location.

### Impact of *PNPLA3* Polymorphisms to the Pathogenicity of NAFLD

We next examined whether or not the seven SNPs in the *PNPLA3* gene were associated with the pathogenic status of NAFLD. The genotype distributions of these SNPs were compared by Jonckheere-Terpstra test among the four subgroups of NAFLD patients categorized by Matteoni’s classification (type1 to type4). There was a significant increase in the frequency of the risk allele from Matteoni type1 to type4 for all of the seven SNPs (*p*-values ranging from 3.6×10^−6^ to 0.0017) (Table 2). Among them, rs738409 again showed the strongest association (*p* = 3.6×10^−6^) as seen in the simple case/control analysis. On the other hand, there was no significant association between control and Matteoni type1 (*p* = 0.76).

**Figure 3:**
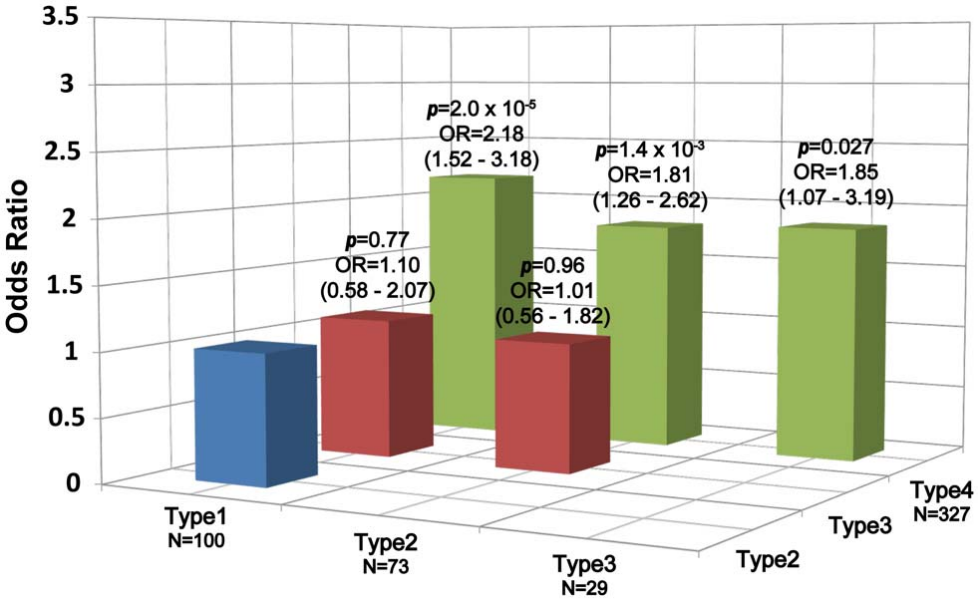
Histogram of odds ratios for genotype distribution of rs738409 between Matteoni types. Each box denotes the odds ratio (OR) comparing the corresponding Matteoni types on the horizontal axes. N represents the number of samples. Odds ratios and *p*-values are calculated for the higher Matteoni type per risk allele (G) on additive model by multivariable logistic regression adjusted for age, sex and BMI, and are shown with 95% CI above each box.

In order to clarify how rs738409 influences the pathogenicity of NAFLD, we performed pairwise comparisons of genotype distributions in the four subgroups of NAFLD patients. There were marked differences in genotype distributions between type4 subgroup and the other three subgroups by multivariable logistic regression adjusted for age, sex and body mass index (BMI) (*p* = 2.0×10^−5^, OR = 2.18, 95%CI: 1.52–3.18 between type1 and type4; *p* = 1.4×10^−3^, OR = 1.81, 95%CI: 1.26–2.62 between type2 and type4; *p* = 0.027, OR = 1.85, 95%CI: 1.07–3.19 between type3 and type4) (Figure 3). On the other hand, no significant associations were obtained for type1 to type3 in any combinations. When we performed the same analysis between type4 and the pooled genotypes of type1 to type3, we again obtained a significant difference (*p* = 4.8×10^−6^, OR = 1.96, 95%CI: 1.47–2.62).

We further examined the specific association of rs738409 with type4 subgroup by using the case/control association results of the initial genome scan. 529 NAFLD patients were divided into 202 patients with type1 to type3 and 327 patients with type4, and genotype distributions of rs738409 in each subgroup were compared with those of 932 control subjects. Exact trend test returned an extremely strong association of rs738409 with type4 subgroup (*p* = 1.7×10^−16^, OR = 2.18, 95%CI: 1.81–2.63) whereas no association was obtained for type1 to type3 subgroups (*p* = 0.41).

### Association of rs738409 Genotypes with Clinical Traits

The quantitative effects of rs738409 genotypes to clinical traits were examined by multivariable regression adjusted for age, sex and BMI (statistical calculation 1, Table 3). Five categorical ordinals, namely, anti-nuclear antibody (ANA), Brunt grade, Brunt stage, fat deposition and iron deposition, were also tested by an ordinal logistic regression analysis. Potential associations (*p*<0.05) were obtained for 11 traits, namely, aspartate transaminase (AST), alanine aminotransferase (ALT), type IV collagen 7S, hyaluronic acid, hemoglobin A1c (HbA1c), fasting immunoreactive insulin (IRI), fasting plasma glucose (FPG), platelet count (PLT), Brunt grade, fat deposition and iron deposition (Table 3). When the results were further adjusted for Matteoni type (statistical calculation 2), AST, hyaluronic acid, HbA1c, FPG, PLT, Brunt grade and iron deposition showed *p*-values smaller than 0.05. The level of serum triglyceride was not significant in the initial analysis, but became significant after being adjusted for Matteoni’s type (*p* = 0.013). Among them, only three traits, namely, hyaluronic acid, HbA1c and iron deposition, remained significant (*p*<0.0021) after Bonferroni’s correction for multiple testing (Table 3).

**Table 3:** Association of rs738409 with clinical traits.

Biochemical traits	Statistical calculation1		Statistical calculation 2	
Phenotype	Coef. (S.E.)	*p*-value	Coef. (S.E.)	*p*-value
Biological traits				
AST (IU/L)	0.22 (0.056)	**1.2×10^−4^**	0.11 (0.052)	0.038
ALT (IU/L)	0.19 (0.058)	**0.0016**	0.093 (0.056)	0.098
GGT (IU/L)	−0.056 (0.061)	0.37	−0.088 (0.062)	0.16
Albumin (g/dL) *	0.015 (0.051)	0.77	−0.012 (0.052)	0.81
Total bilirubin (mg/dL)	−0.011 (0.063)	0.86	0.0059 (0.064)	0.93
Cholinesterase (unit) *	0.062 (0.040)	0.12	0.069 (0.041)	0.092
Type IV collagen 7S (ng/dL) *	−0.19 (0.064)	0.0025	−0.11 (0.062)	0.069
Hyaluronic acid (ng/dL)	0.30 (0.065)	**4.9×10^−6^**	0.22 (0.063)	**4.6×10^−4^**
Triglycerides (mg/dL)	−0.10 (0.058)	0.072	−0.15 (0.059)	0.013
Total cholesterol (mg/dL)	−0.066 (0.060)	0.27	−0.057 (0.061)	0.34
HbA1c (%)	−0.17 (0.053)	**0.0012**	−0.18 (0.054)	**0.0011**
IRI (µg/dL)	0.16 (0.063)	0.012	0.086 (0.061)	0.16
FPG (mg/dL)	−0.14 (0.049)	0.0047	−0.15 (0.05)	0.0035
HOMA-IR	0.084 (0.064)	0.19	0.0092 (0.062)	0.88
Hs-CRP (mg/dL)	−0.013 (0.048)	0.79	−0.031 (0.049)	0.52
Adiponectin (µg/mL)	0.048 (0.066)	0.47	0.12 (0.066)	0.072
Leptin (ng/mL)	0.11 (0.068)	0.11	0.10 (0.069)	0.15
Ferritin (ng/mL)	0.031 (0.047)	0.51	−0.0042 (0.048)	0.93
Uric acid (mg/dL)	−0.097 (0.061)	0.11	−0.11 (0.062)	0.067
PLT (x10^4^/µL)	−0.056 (0.020)	0.0052	−0.045 (0.020)	0.028
Immunological/histological traits				
ANA (0/1/2/3/4)	0.92 (0.70–1.21)	0.56	0.86 (0.65–1.15)	0.31
Brunt grade (1/2/3)	1.42 (1.06–1.92)	0.021	1.38 (1.02–1.87)	0.036
Brunt stage (1/2/3/4)	1.28 (0.95–1.72)	0.11		
Fat deposition (1/2/3/4)	1.44 (1.15–1.81)	0.0019	1.24 (0.98–1.56)	0.76
Iron deposition (0/1/2/3/4)	0.61 (0.47–0.80)	**3.0×10^−4^**	0.62 (0.47–0.81)	**5.6×10^−4^**

Associations between distribution of rs738409 genotypes and clinical traits are calculated by multivariable regression. Statistical calculation1 is adjusted for age, sex and BMI, while the Matteoni types are additionally included as covariate in statistical calculation 2. Statistics are calculated by multivariable linear regression for biochemical traits and by multivariable ordinal logistic regression for immunological and histological traits.

Coefficients and odds ratios are calculated for the increase of each trait per risk allele (G). The *p*-values showing significance after Bonferroni’s correction for multiple testing (*p* = 0.0021) was shown in bold.

* Reciprocal numbers are used for normalization and a negative coefficient implicates an increase in value according to the increase of the risk allele.

### Associations of Previously Reported SNPs with NAFLD

Previous genetic studies identified four chromosomal loci, namely, *LYPLAL1* at 1q41, *GCKR* at 2p23, *NCAN* at 19p12 and *PPP1R3B* at 8p23.1, associated with NAFLD in populations of European descent [14]. We examined whether or not the associations were reproduced in the Japanese population by extracting genotype information of SNP markers corresponding to these four loci. As shown in Table 4, the association of rs780094 in *GCKR* with NAFLD was at the border of significance (*p* = 0.011, OR = 0.82, 95%CI: 0.70–0.91) in the case/control analysis. However, the association was lost when examined between rs780094 genotypes and Matteoni types. There were no associations of rs2228603 in *NCAN* and rs12137855 in *LYPLAL1* with either NAFLD or Matteoni types. Rs4240624 in *PPP1R3B* was not in the SNP array used for this study, and this marker was not polymorphic or at a very low frequency in the Japanese (0 in 90 chromosomes in the Japanese result of the International HapMap Project).

## Discussion

NASH is a type of hepatic steatosis in NAFLD with poor prognosis accompanying liver fibrosis, and subsequent liver cirrhosis and hepatocellular carcinoma [18]. Despite the extensive biochemical and histological investigation of NAFLD, whether or not NASH forms a distinct disease entity in NAFLD still remains unclear. The principle aim of this study was to identify the genetic factors related to the pathogenic status of NAFLD by collecting DNA samples of Japanese NAFLD patients with critically diagnosed disease status by liver biopsy. To our knowledge, this is the first GWA study of NAFLD using patients with known histology-based Matteoni type. In the initial association study using pooled genotyping results of all the cases, we found a significant association of the *PNPLA3* gene at chromosome 22q13.31 with NAFLD in the Japanese. Rs738409 which showed the strongest association with NAFLD in the GWA study of Caucasians was also genotyped and its strongest association with NAFLD was confirmed. These results were in agreement with the former GWA analyses in populations of European descent and in Hispanics, giving strong evidence of the involvement of *PNPLA3* in NAFLD beyond ethnicities. Rs738409 is located in exon3 of the *PNPLA3* gene which is expressed in the liver and adipose tissue. This SNP introduces an amino acid substitution from isoleucine to methionine (I148M), and biological studies demonstrated that its risk allele (G) abolishes the triglyceride hydrolysis activity of *PNPLA3* [19]. These observations strongly suggest rs738409 to be a causative genetic variation for NAFLD. However, future genomic analyses by fine mapping or extensive sequencing may identify additional genetic determinants within the *PNPLA3* locus.

In the current study we did not find other genetic loci showing genome-wide significance (*p*<1.0×10^−7^). However, two additional chromosomal loci with *p*-values being smaller than 1×10^−5^ were identified on chromosome 1p (rs11206226) and chromosome 4p (rs1390096) neither of which has been reported as being associated with NAFLD in Caucasians (Table S1). Statistical calculation by taking their allele frequencies and effect sizes into account showed that approximately three times as many case and control samples are required to obtain sufficient statistical power (>0.8) for genome wide significance. Hence, further confirmation is required using a larger collection of patients and controls although they may be potential candidates of low-penetrance genes for susceptibility to NAFLD in Japanese.

Subsequent analyses through comparison of genotype distribution among four subgroups of NAFLD (type1 to type4) categorized by Matteoni’s classification revealed that the seven NAFLD-associated SNPs in the *PNPLA3* gene were also significantly associated with the pathogenic status of NAFLD. There were also marked differences in genotype distribution of rs738409 between type4 subgroup and the other three groups (*p* = 4.8×10^−6^, OR = 1.96, 95%CI: 1.47–2.62 between type4 and pooled genotypes of type1 to type3). Moreover, a case/control analysis of rs738409 between Matteoni type4 and controls returned a surprisingly strong association (*p* = 1.7×10^−16^) which was much stronger than the initial analysis using all NAFLD cases (*p* = 1.4×10^−10^), whereas the analysis using Matteoni type1 to type3 as cases didn’t show significance (*p* = 0.41). There were differences in the score of HOMA-IR and hs-CRP, indicators of insulin resistance and inflammation, respectively, between Matteoni type1 to type3 and type4 subgroups (Table 1). Our results provide compelling evidence that NASH corresponding to Matteoni type4 is both a clinically and genetically different disease subset from other spectrums of NAFLD. Previous studies showed association between *PNPLA3* and fatty liver, inflammation, fibrosis grade and NASH [13]. In our result, strong association between rs738409 and fatty liver was not observed by comparing control and Matteoni type1. In addition, strong association between rs738409 and lobular inflammation was not observed by comparing Matteoni Type1 and Type2. In contrast, a strong association between rs738409 and NASH was observed. Although we could not observe the strong association between rs738409 and fibrosis stage, strong association between rs738409 and Hyaluronic acid suggests that an association exists between *PNPLA3* and fibrosis.

We have also undertaken association analyses of rs738409 and clinical traits in the patients. The multivariable regression analysis adjusted for age, sex, BMI and Matteoni type followed by the correction for multiple testing revealed hyaluronic acid and HbA1c as being significantly associated with rs738409. Hyaluronic acid is one of the principle components of the extracellular matrix and its involvement in fibrosis has been previously suggested [20]. This may indicate another possible functional involvement of *PNPLA3* in the progression of liver fibrosis by influencing the circulating hyaluronic acid levels. A weak association of rs738409 and HbA1c levels was observed in our study population. However, there are no reports to date indicating such an association, and confirmation with different sample sets is needed for definitive conclusion. Also, the association between rs738409 and iron deposition was demonstrated by an ordinal logistic regression analysis. Since the association still remained after the results were adjusted with Matteoni type, rs738409 may play a functional role in the oxidative stress through iron absorption in the liver.

Recently, a genetic analysis of Japanese NAFLD patients was reported demonstrating a significant association in the increase of AST, ALT, ferritin levels and fibrosis stage (Brunt stage) and in the decrease of serum triglyceride with the risk allele (G) of rs738409 [12]. In our study, the association of rs738409 with AST (*p* = 1.2×10^−4^) and ALT (*p* = 0.0016) was reproduced and that of AST still remained after the results were adjusted for Matteoni type (*p* = 0.038). No association was observed for ferritin level. Brunt stage was available for Matteoni type4 patients only in our study. Although the odds ratio was slightly high (OR = 1.28, 95%CI: 0.95–1.72), it was not possible to examine the association. In addition, the inverse association of the risk allele of rs738409 with decrease of serum triglyceride was confirmed in our study (*p* = 0.013 after being adjusted for Matteoni type). For all of these biomarkers, however, the significance was lost after the correction for multiple testing.

A replication analysis of other genetic loci that had been reported for their association with NAFLD in East coast white Americans [14] was performed in our sample collection. We confirmed the association of rs780094 in *GCKR* with NAFLD in a case/control analysis but at a much weaker level (*p* = 0.011, OR = 0.82, 95%CI: 0.70–0.95) than that shown for the populations of European-descent. No associations were found for *LYPLAL1* and *NCAN* loci in our study. There are several reasons to explain such differences, such as the insufficient statistical power with a limited number of study subjects in our study due to the difficulty in the collection of a larger number of histologically diagnosed NAFLD patients. The difference in genetic background between the Japanese and Europeans is also conceivable. Indeed, the risk allele frequency of rs12137855 in *LYPLAL1* was 0.944 in our control subjects but approximately 0.79 in the European populations [14]. Similarly, there was a difference in the risk allele frequency of rs2228603 in *NCAN* (0.049 in Japanese and 0.08 in Europeans). Rs4240624 in *PPP1R3B* was not polymorphic in the Japanese while its risk allele frequency was 0.91 in Europeans.

## Materials and Methods

### Ethics Statement

In compliance with the Declaration of Helsinki, ethical approval for this study was given by the respective Institutional Review Board and subject written informed consent were obtained for all subjects (Ethical committee of Nara City Hospital; Ethical committee of Saiseikai Suita Hospital; Medical Ethics Committee of Kanazawa University; Ethics committee of Kyoto Prefectural University of Medicine; Ethical Committee of Aichi Cancer Center; Ethical Committee of Kochi Medical School, Kochi University; Ethics Committee of Tokyo Women’s Medical University; Ethical Committee on Kawasaki Medical School and Kawasaki Medical School Hospital; Ethical Committee of Juntendo University; Ethics Committee of Yamagata University School of Medicine; Ethical Committee of the Ikeda Municipal Hospital; Institutional Review Board and Ethics Committee of Kyoto University School of Medicine).

### Study Population

A total of 543 patients histologically diagnosed for NAFLD in 2007–2009 were recruited through the Japan study of Nonalcoholic Fatty Liver Disease. Biopsy specimens were stained with H&E and Masson’s trichrome for morphological review and assessment of fibrosis. Perl’s Prussian blue was performed to evaluate iron load. Biopsy specimens were reviewed by a hepatopathologist (T.O). NAFLD patients were classified into four categories by liver histology according to the classification by Matteoni *et al* [2] as follows; type1: fatty liver alone, type2: fat accumulation and lobular inflammation, type3: fat accumulation and ballooning degeneration, type4: fat accumulation, ballooning degeneration, and either Mallory-Denk body or fibrosis. With these criteria, the 543 patients were classified as type1; 102, type2; 75, type3; 31 and type4; 335. The histological grade and fibrosis stage were also evaluated by the classification of Brunt *et al* [21] for advanced NAFLD cases (type3 and type4) as follows; grade 1: steatosis involving up to 66% of biopsy, occasional ballooned zone 3 hepatocytes and absence or mild portal chronic inflammation, grade2: steatosis, ballooning hepatocytes mild to moderate chronic inflammation, grade3: panacinar steatosis, ballooning and disarray obvious and mild or portal mild to moderate inflammation, stage1: perivenular and/or perisinusoidal fibrosis in zone3, stage2: combined pericellular portal fibrosis, stage3: septal/bridging fibrosis, stage4: cirrhosis. The degree of fat deposition was evaluated by amount of fat droplets as observed under the microscope as follows; 0: <5%, 1: 5–<10%, 2: 10–<34%, 3: 34–<67%, 4: >67%. The degree of iron deposition was categorized by the presence of granules of free iron observed under the microscope as follows; 0: absence by x400, 1: easily identifiable by x400 and rarely identifiable by x250, 2: identifiable by x100, 3: identifiable by x25, 4: identifiable at lower than x25.

Inclusion criteria for NAFLD patients were as follows; (i) no history of alcoholism, (ii) no history for HBV/HCV/HIV infection, (iii) diagnosed by liver biopsy, (iv) information regarding age and BMI available. The sex of two samples was unknown, and was imputed from the results of the genome scan. As general Japanese population controls, the genome scan results of 942 healthy Japanese volunteers from Aichi Cancer Center Hospital and Research Institute were used [22].

### Anthropometric and Laboratory Evaluation

We employed conventional methods for the measurement of anthropometry (height, weight, amount of visceral fat and abdominal circumscript). BMI was calculated from the measurements. The following biochemical/hematological/immunological traits were also measured by conventional methods; aspartate aminotransferase (AST), alanine aminotransferase (ALT), γ-glutamyl transpeptidase (GGT), albumin, total bilirubin, cholinesterase, type IV collagen 7S, hyaluronic acid, triglyceride, total cholesterol, hemoglobin A1c (HbA1c), fasting immunoreactive insulin (IRI), fasting plasma glucose (FBS), high sensitive CRP (hs-CRP), adiponectin, leptin, ferritin, uric acid, and platelet (PLT) count. Anti nuclear antibody (ANA) was measured by ELISA and categorized by the detection limit in a serial dilution as follows; 0: <40x, 1: 40–80x, 2: 81–160x, 3: 160x, 4: >320x. Homeostasis model assessment-insulin resistance (HOMA-IR) was calculated from the measurements. Patients were assigned a diagnosis of diabetes mellitus (DM) when they had documented use of oral hypoglycemic medication, a random glucose level >200 mg/dl, or FPG >126 mg/dl. Hyperlipidemia was diagnosed with the cholesterol level being >200 mg/dl and/or triglyceride level being >160 mg/dl. Hypertension was diagnosed when the patient was taking antihypertensive medication and/or had a resting recumbent blood pressure ≧140/90 mmHg on at least two occasions.

### DNA Preparation

Genomic DNA was extracted from peripheral blood mononuclear cells by standard phenol-chloroform extraction and resuspended in TE buffer. DNA concentration and purity were measured with Nanodrop 1000 spectrophotometer (Thermo Scientific, Waltham, MA, USA). The samples were stored at −20°C until use.

**Table 4:** Replication study of previously reported SNPs.

			Genotyping Result and Allele Frequency of A2					Statistics		
			NAFLD					NAFLD vs. Control		Matteoni
dbSNPID	A1/A2	Gene	Control	Type 1	Type 2	Type 3	Type 4	*p-*value†	OR (95%CI)	*p-*value‡
rs12137855	C*/T	*LYPLAL1*	828/102/2	90/10/0	67/6/0	24/5/0	294/33/0	0.55	0.89	0.98
			(0.056)	(0.050)	(0.041)	(0.086)	(0.050)		(0.64–1.25)	
rs780094	T*/C	*GCKR*	321/433/178	34/54/12	28/34/11	17/11/1	133/139/55	0.011	0.82	0.92
			(0.423)	(0.390)	(0.383)	(0.224)	(0.381)		(0.70–0.95)	
rs4240624	G/A	*PPP1R3B*	–	–	–	–	–	–	–	–
rs2228603	C/T*	*NCAN*	842/88/2	93/7/0	65/8/0	28/1/0	292/31/4	0.80	1.05	0.58
			(0.049)	(0.035)	(0.054)	(0.017)	(0.059)		(0.75–1.48)	

Reference (A1) and non-reference (A2) alleles refer to NCBI Reference Sequence Build 36.3 with the effective allele marked by an asterisk. Genotyping results are shown by genotype count of A1A1/A1A2/A2A2 with allele frequency of A2 in parenthesis. †*P*-values are calculated by exact trend test with odds ratios (OR) calculated for A2 with 95% confidence interval (CI). ‡*P*-values are calculated by Jonckheere-Terpstra test in NAFLD patients for Matteoni type and additive model of genotype.

### Genome-wide Genotyping and Quality Control

Genome scan was conducted for 543 patients with NAFLD and 942 healthy subjects using Illumina Human 610-Quad Bead Chip on a Bead Station 500G Genotyping System (Illumina, Inc., San Diego, CA, USA) and subjected to the following quality controls. Initially, ten patients and six control subjects were removed due to low call rates (<0.99). Regarding the SNP markers, 85,472 SNPs with minor allele frequency of smaller than 0.01 in either case or control group, 6,479 SNPs with lower success rates (<0.98) and 35 SNPs with distorted Hardy-Weinberg equilibrium (p<10^−7^) were removed, resulting in 484,751 SNP markers being used for analysis. Principal component analysis by EIGENSOFT [17] including phase II HapMap (http://hapmap.ncbi.nlm.nih.gov/) samples identified no samples that were deviated from the Japanese population. Subsequently, the degree of kinship between individuals was examined by pi-hat in PLINK 1.07 (http://pngu.mgh.harvard.edu/purcell/plink/) [23]. Of the eight pairs of samples (four case pairs and four control pairs) showing high degrees of kinship (PI-HAT>0.4), the sample with the lower call rate in each pair was removed. After these steps, 529 case and 932 controls were used for the analysis.

### Statistical Analysis

A case/control association analysis was performed by exact trend test between NAFLD patients and control subjects [24]. The correction of obtained *p*-values for population stratification was performed using EIGENSTRAT [17] **.** In addition, an association between Matteoni classification (type1 to type4) and additive model of genotype for each SNP was examined using Jonckheere-Terpstra test for NAFLD patients. Assessment of population stratification of inflation of *p-*value was carried out by the genomic control method for asymptotic trend test [25]. Association between each quantitative trait and the genotype of significant SNPs in NAFLD patients were calculated by multivariable linear regression or multivariable ordinal regression adjusted for age, sex and BMI. Each quantitative trait was transformed as follows; natural log for ALT, AST, HOMA-IR, HbA1c, IRI, triglyceride, total bilirubin, adiponectin, hs-CRP, hyaluronic acid, leptin, reciprocal number for albumin, cholinesterase, type IV collagen 7S and square root for uric acid and ferritin. The values of FPG, PLT, total cholesterol, amount of visceral fat, and abdominal circumscript were not transformed. For each trait, values that were within only 4 S.D. were included for analysis. LD indices were calculated by default setting of Haploview [26] and the LD block was defined manually.

## Supporting Information

Figure S1 **QQ plot of the GWA study comparing distribution of the observed and expected** ***p*** **-values.** Upper box is expressed in antilog scale and the lower box is expressed in –log_10_ scale. The X- and Y-axis correspond to expected and observed *p*-values. Blue and red dots denote before and after correction by genomic control method (λ = 1.04), respectively.
(DOC)

Table S1 **List of the SNPs showing** ***p*** **<1.0×10^−5^ in the GWA study.** Reference (A1) and non-reference (A2) alleles refer to NCBI Reference Sequence Build 36.3 with the effective allele marked by an asterisk. Genotyping results are shown by genotype count of A1A1/A1A2/A2A2 with allele frequency of A2 in parenthesis. †*P*-values are calculated by exact trend test with odds ratios (OR) calculated for A2 with 95% confidence interval (CI). ‡*P*-values are calculated by Jonckheere-Terpstra test in NAFLD patients for Matteoni type and additive model of genotype. SNPs are ordered by chromosomal location.
(DOC)

## References

[1] Ludwig J, Viggiano TR, McGill DB, Oh BJ (1980). Nonalcoholic steatohepatitis: Mayo Clinic experiences with a hitherto unnamed disease.. Mayo Clin Proc.

[2] Matteoni CA, Younossi ZM, Gramlich T, Boparai N, Liu YC (1999). Nonalcoholic fatty liver disease: a spectrum of clinical and pathological severity.. Gastroenterology.

[3] Cohen JC, Horton JD, Hobbs HH (2011). Human fatty liver disease: old questions and new insights. Science 332: 1519–1523.. doi:10.1126/science.1204265.

[4] Vernon G, Baranova A, Younossi ZM (2011). Systematic review: the epidemiology and natural history of non-alcoholic fatty liver disease and non-alcoholic steatohepatitis in adults. Aliment Pharmacol Ther 34: 274–285.. doi.

[5] Okanoue T, Umemura A, Yasui K, Itoh Y (2011). Nonalcoholic fatty liver disease and nonalcoholic steatohepatitis in Japan. J Gastroenterol Hepatol 26 Suppl 1: 153–162.. doi.

[6] Williams CD, Stengel J, Asike MI, Torres DM, Shaw J (2011). Prevalence of nonalcoholic fatty liver disease and nonalcoholic steatohepatitis among a largely middle-aged population utilizing ultrasound and liver biopsy: a prospective study. Gastroenterology 140: 124–131.. doi:10.1053/j.gastro.2010.09.038.

[7] Okanoue T (2011). Recent progress in the research of NASH/NAFLD in Japan.. Nihon Shokakibyo Gakkai Zasshi.

[8] Berson A, De Beco V, Lettéron P, Robin MA, Moreau C (1998). Steatohepatitis-inducing drugs cause mitochondrial dysfunction and lipid peroxidation in rat hepatocytes.. Gastroenterology.

[9] Day CP (2006). From fat to inflammation. Gastroenterology 130: 207–210.. doi:10.1053/j.gastro.2005.11.017.

[10] Romeo S, Kozlitina J, Xing C, Pertsemlidis A, Cox D (2008). Genetic variation in PNPLA3 confers susceptibility to nonalcoholic fatty liver disease. Nat Genet 40: 1461–1465.. doi:10.1038/ng.257.

[11] Sookoian S, Castaño GO, Burgueño AL, Gianotti TF, Rosselli MS (2009). A nonsynonymous gene variant in the adiponutrin gene is associated with nonalcoholic fatty liver disease severity. J Lipid Res 50: 2111–2116.. doi.

[12] Hotta K, Yoneda M, Hyogo H, Ochi H, Mizusawa S (2010). Association of the rs738409 polymorphism in PNPLA3 with liver damage and the development of nonalcoholic fatty liver disease. BMC Med Genet 11: 172.. doi.

[13] Sookoian S, Pirola CJ (2011). Meta-analysis of the influence of I148M variant of patatin-like phospholipase domain containing 3 gene (PNPLA3) on the susceptibility and histological severity of nonalcoholic fatty liver disease. Hepatology 53: 1883–1894.. doi:10.1002/hep.24283.

[14] Speliotes EK, Yerges-Armstrong LM, Wu J, Hernaez R, Kim LJ (2011). Genome-wide association analysis identifies variants associated with nonalcoholic Fatty liver disease that have distinct effects on metabolic traits. PLoS Genet 7: e1001324.. doi:10.1371/journal.pgen.1001324.

[15] Petersen KF, Dufour S, Hariri A, Nelson-Williams C, Foo JN (2010). Apolipoprotein C3 gene variants in nonalcoholic fatty liver disease. N Engl J Med 362: 1082–1089.. doi:10.1056/NEJMoa0907295.

[16] Terao C, Yamada R, Ohmura K, Takahashi M, Kawaguchi T (2011). The human AIRE gene at chromosome 21q22 is a genetic determinant for the predisposition to rheumatoid arthritis in Japanese population. Human Molecular Genetics 20: 2680–2685.. doi:10.1093/hmg/ddr161.

[17] Price AL, Patterson NJ, Plenge RM, Weinblatt ME, Shadick NA (2006). Principal components analysis corrects for stratification in genome-wide association studies. Nat Genet 38: 904–909.. doi:10.1038/ng1847.

[18] Yasui K, Hashimoto E, Komorizono Y, Koike K, Arii S (2011). Characteristics of patients with nonalcoholic steatohepatitis who develop hepatocellular carcinoma. Clin Gastroenterol Hepatol 9: 428–433; quiz e50.. doi:10.1016/j.cgh.2011.01.023.

[19] He S, McPhaul C, Li JZ, Garuti R, Kinch L (2010). A Sequence Variation (I148M) in PNPLA3 Associated with Nonalcoholic Fatty Liver Disease Disrupts Triglyceride Hydrolysis. J Biol Chem 285: 6706–6715.. doi:10.1074/jbc.M109.064501.

[20] Ueno T, Inuzuka S, Torimura T, Tamaki S, Koh H (1993). Serum hyaluronate reflects hepatic sinusoidal capillarization.. Gastroenterology.

[21] Brunt EM, Janney CG, Di Bisceglie AM, Neuschwander-Tetri BA, Bacon BR (1999). Nonalcoholic steatohepatitis: a proposal for grading and staging the histological lesions. Am J Gastroenterol 94: 2467–2474.. doi.

[22] Suzuki T, Matsuo K, Sawaki A, Mizuno N, Hiraki A (2008). Alcohol Drinking and One-Carbon Metabolism-Related Gene Polymorphisms on Pancreatic Cancer Risk. Cancer Epidemiology Biomarkers & Prevention 17: 2742–2747.. doi.

[23] Purcell S, Neale B, Todd-Brown K, Thomas L, Ferreira MAR (2007). PLINK: A Tool Set for Whole-Genome Association and Population-Based Linkage Analyses.. Am J Hum Genet.

[24] Yamada R, Okada Y (2009). An optimal dose-effect mode trend test for SNP genotype tables. Genet Epidemiol 33: 114–127.. doi:10.1002/gepi.20362.

[25] Devlin B, Roeder K (1999). Genomic control for association studies.. Biometrics.

[26] Barrett JC, Fry B, Maller J, Daly MJ (2005). Haploview: analysis and visualization of LD and haplotype maps. Bioinformatics 21: 263–265.. doi:10.1093/bioinformatics/bth457.

