# Supplementary material for: Genetic Polymorphisms of the Human PNPLA3 Gene Are Strongly Associated with Severity of Non-Alcoholic Fatty Liver Disease in Japanese
Source: PLoS One. 2012 Jun 14;7(6):e38322. doi: 10.1371/journal.pone.0038322 (PMC3375283; doi:10.1371/journal.pone.0038322)
Supplement: Figure S1 — QQ plot of the GWA study comparing distribution of the observed and expected p -values. Upper box is expressed in antilog scale and the lower box is expressed in –log10 scale. The X- and Y-axis correspond to expected and observed p-values. Blue and red dots denote before and after correction by genomic control method (λ = 1.04), respectively. (DOC) [file pone.0038322.s001.doc]

**Figure S1. QQ plot of the GWA study comparing distribution of the observed and expected *p*-values.**

**
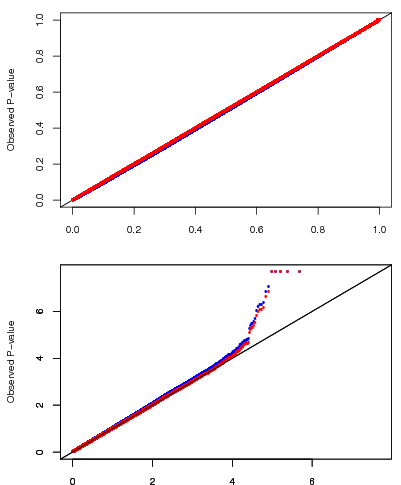
**

Upper box is expressed in antilog scale and the lower box is expressed in –log_10_ scale. The X- and Y-axis correspond to expected and observed *p*-values. Blue and red dots denote before and after correction by genomic control method (λ=1.04), respectively.
