# Supplementary material for: Genetic Polymorphisms of the Human PNPLA3 Gene Are Strongly Associated with Severity of Non-Alcoholic Fatty Liver Disease in Japanese
Source: PLoS One. 2012 Jun 14;7(6):e38322. doi: 10.1371/journal.pone.0038322 (PMC3375283; doi:10.1371/journal.pone.0038322)
Supplement: Table S1 — List of the SNPs showing p <1.0×10^−5^ in the GWA study. Reference (A1) and non-reference (A2) alleles refer to NCBI Reference Sequence Build 36.3 with the effective allele marked by an asterisk. Genotyping results are shown by genotype count of A1A1/A1A2/A2A2 with allele frequency of A2 in parenthesis. †P-values are calculated by exact trend test with odds ratios (OR) calculated for A2 with 95% confidence interval (CI). ‡P-values are calculated by Jonckheere-Terpstra test in NAFLD patients for Matteoni type and additive model of genotype. SNPs are ordered by chromosomal location. (DOC) [file pone.0038322.s002.doc]

**Table S1. List of the SNPs showing *p* < 1.0 x 10^-5^ in the GWA study**

| dbSNPID | Chr | Allele | | Gene | Genotyping result | | | | | |  | Statistics | | | |
| --- | --- | --- | --- | --- | --- | --- | --- | --- | --- | --- | --- | --- | --- | --- | --- |
|  |  | A1 | A2 |  | Control | NAFLD | | | | |  | NAFLD vs. Control | |  | Matteoni |
|  |  |  |  |  |  | Total | Type 1 | Type 2 | Type 3 | Type 4 |  | *p-*value† | OR (95%CI) |  | *p-*value‡ |
| rs11206226 | 1 | A* | G | *YIPF1* | 667/247/18 (0.152) | 433/93/3 (0.094) | 84/16/0 (0.080) | 63/10/0 (0.068) | 23/6/0 (0.103) | 263/61/3 (0.102) |  | 4.5 x 10^-6^ | 0.58 (0.45-0.74) |  | 0.45 |
| rs1390096 | 4 | G | A* | *HS3ST1* | 358/454/120 (0.372) | 155/260/114 (0.461) | 29/47/24 (0.475) | 17/42/14 (0.479) | 10/12/7 (0.448) | 99/159/69 (0.454) |  | 2.5 x 10^-6^ | 1.44 (1.24-1.68) |  | 0.56 |
| rs738491 | 22 | C | T* | *SAMM50* | 193/468/271 (0.542) | 76/239/214 (0.630) | 15/59/26 (0.555) | 15/32/26 (0.575) | 6/8/15 (0.655) | 40/140/147 (0.664) |  | 3.9 x 10^-6^ | 1.44 (1.24-1.68) |  | 0.0048 |
| rs2073082 | 22 | G* | A | *SAMM50* | 404/421/107 (0.341) | 299/193/37 (0.252) | 49/44/7 (0.290) | 35/28/10 (0.329) | 19/7/3 (0.224) | 196/114/17 (0.226) |  | 8.0 x 10^-7^ | 0.65 (0.55-0.77) |  | 0.0013 |
| rs3761472 | 22 | A | G* | *SAMM50* | 276/461/195 (0.457) | 108/258/162 (0.551) | 25/56/18 (0.465) | 22/32/19 (0.479) | 7/14/8 (0.517) | 54/156/117 (0.596) |  | 1.1 x 10^-6^ | 1.46 (1.26-1.70) |  | 8.4 x 10^-4^ |
| rs2235776 | 22 | C | T* | *SAMM50* | 275/463/194 (0.457) | 108/258/163 (0.552) | 25/56/19 (0.470) | 22/32/19 (0.479) | 7/14/8 (0.517) | 54/156/117 (0.596) |  | 9.2 x 10^-7^ | 1.47 (1.26-1.71) |  | 7.3 x 10^-4^ |
| rs2143571 | 22 | G | A* | *SAMM50* | 272/466/194 (0.458) | 109/253/167 (0.555) | 26/53/21 (0.475) | 22/32/19 (0.479) | 7/13/9 (0.534) | 54/155/118 (0.598) |  | 6.4 x 10^-7^ | 1.47 (1.27-1.72) |  | 0.0011 |
| rs6006473 | 22 | C | T* | *SAMM50* | 189/471/272 (0.545) | 76/236/217 (0.633) | 15/59/26 (0.555) | 15/32/26 (0.575) | 6/8/15 (0.655) | 40/137/150 (0.668) |  | 3.1 x 10^-6^ | 1.44 (1.24-1.69) |  | 0.0031 |
| rs2073080 | 22 | C | T* | *PARVB* | 272/466/194 (0.458) | 109/254/166 (0.554) | 26/53/21 (0.475) | 22/32/19 (0.479) | 7/13/9 (0.534) | 54/156/117 (0.596) |  | 7.8 x 10^-7^ | 1.47 (1.26-1.71) |  | 0.039 |
| rs2281292 | 22 | A | C* | *PARVB* | 188/472/272 (0.545) | 76/235/218 (0.634) | 15/58/27 (0.560) | 15/32/26 (0.575) | 6/8/15 (0.655) | 40/137/150 (0.668) |  | 3.1 x 10^-6^ | 1.45 (1.24-1.69) |  | 0.0043 |

Reference (A1) and non-reference (A2) alleles refer to NCBI Reference Sequence Build 36.3 with the effective allele marked by an asterisk. Genotyping results are shown by genotype count of A1A1/A1A2/A2A2 with allele frequency of A2 in parenthesis. †*P*-values are calculated by exact trend test with odds ratios (OR) calculated for A2 with 95% confidence interval (CI). ‡*P*-values are calculated by Jonckheere-Terpstra test in NAFLD patients for Matteoni type and additive model of genotype. SNPs are ordered by chromosomal location.
